# Low-pressure plasma cleaning of organic contamination from chemically coated surfaces: a study combining experimental and molecular dynamics studies

**DOI:** 10.1039/d5ra05699c

**Published:** 2025-11-18

**Authors:** Zixu Wang, Yuhai Li, Peng Zhang, Fei Wang, Laixi Sun, Qingshun Bai, Mingzhi Zhu, Baoxu Wang

**Affiliations:** a Institute of Systems Engineering, China Academy of Engineering Physics Mianyang 621999 China; b Laser Fusion Research Center, China Academy of Engineering Physics Mianyang 621900 China; c School of Mechatronics Engineering, Harbin Institute of Technology Harbin 150000 China; d Chongqing Research Institute, Harbin Institute of Technology Chongqing 401135 China

## Abstract

During prolonged service in vacuum-based intense laser systems, the surface chemical coatings of large-aperture optical components inevitably suffer from organic contamination, leading to irreversible damage to the surface chemical coatings and a rapid degradation of the optical performance under laser irradiation. This study uses experiments and simulations to examine the process and microscopic mechanisms associated with the removal of organic contaminants from surface chemical coatings through low-pressure plasma cleaning. Firstly, a capacitive-coupling discharge model for the low-pressure plasma cleaning device was constructed through finite element simulations to obtain the spatial distribution of plasma discharge characteristics. Langmuir probe and emission spectrometer experiments were conducted to explore the plasma discharge laws and determine the effects of the plasma parameters on the discharge characteristics and the types of reactive particles excited in low-pressure plasma of oxygen and argon gas. Secondly, a quantitative relationship between the number of typical functional groups in organic contaminants and the transmittance of optical components was established. Various low-pressure plasma cleaning experiments were performed by adjusting the core plasma parameters to analyze the effects of these parameters on the cleaning performance. Finally, based on the reactive force field, a molecular dynamics model of the interaction between the plasma and organic contaminants was constructed. Simulation results revealed the reaction mechanisms between the plasma and organic contaminants, which provided a theoretical explanation of the microscopic mechanism of plasma cleaning. The results of this study offer theoretical guidance and practical verification from the microscopic mechanism and macroscopic practical perspective for the efficiency improvement of the plasma cleaning technology for organic contaminants.

## Abstract 2

Low-pressure oxygen plasma removes realistic organic films from coated optics *via* radical-driven pathways. Probe/Raman spectroscopy and reactive MD converge on process windows that restore near-baseline optical performance.

## Graphical abstract

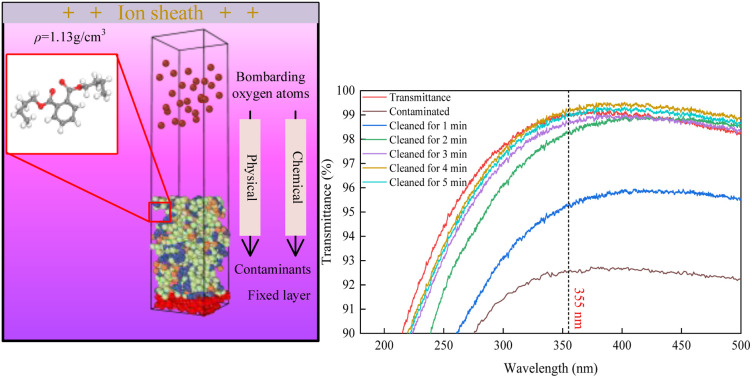

## 1. Introduction

Due to the unique characteristics in the frequency, time, and energy domains, the high-performance laser output from intense laser facilities has become an indispensable tool in cutting-edge research areas such as nuclear physics, atomic physics, modern manufacturing, and fundamental scientific studies.^1,2^ To enhance the output capability of intense laser facilities, core large-aperture optical components are often coated with chemical coatings, such as anti-reflective coatings, high-reflective coatings, optical filters, and beam-splitting coatings, to improve the optical performance of the components and increase their durability and stability.^3–5^ The energy output of intense laser facilities is primarily limited by the damage to the chemical coatings and the optical components themselves, caused by the surface contamination of large-aperture optical components.^6^ Surface contaminants on optical components in intense laser systems primarily include particulate contaminants, organic contaminants, and moisture. The effective control of particulate and moisture contaminations has been largely achieved through methods such as negative-pressure cycling, air-knife purging, and temperature-regulated techniques.^7^ However, the presence of organic contaminants on large-aperture optical components persists as a critical unresolved issue during prolonged system operation. Free organic contaminants continuously deposit onto the chemical coatings on the surfaces of optical components in the vacuum environment during prolonged operation of the laser facility. Under the irradiation of intense lasers, the organic contaminants undergo ablation or decomposition, generating stray light, which leads to the damage and detachment of the chemical coatings on the optical components' surfaces. The damage of the chemical coatings reduces the optical components' damage threshold, limiting the operational efficiency of the intense laser system.^8–10^ Experimental results have demonstrated that the contamination of the optical component surfaces can induce damage spots five times the size of the contaminants themselves under the irradiation of intense lasers, leading to a reduction of approximately 60% in the laser damage threshold of the optical components.^11^ Currently, various cleaning methods have been developed for the removal of contaminants from the chemical coatings on the surfaces of large-aperture optical components, including dry cleaning (*e.g.*, ultraviolet ozone cleaning, laser cleaning) and wet cleaning (*e.g.*, organic solvent cleaning, piranha solution cleaning, ultrasonic cleaning). However, due to the difficulty of disassembling and transporting large-aperture optical components in inertial confinement fusion (ICF) facilities, the high precision required during installation, and the ongoing contamination of optical component surfaces during extended operation, cleaning techniques for optical components are subject to higher demands. Low-pressure plasma cleaning technology can ionize the working gas *via* low-pressure radio-frequency (RF) capacitive coupling discharge, generating a large-area, uniform, diffuse plasma with randomly directed ion bombardment under relatively low pressure and temperature conditions.^12–15^ This technology can efficiently and non-destructively clean optical components with chemical coatings with large dimensions, complex structures, and high cleanliness requirements without causing secondary contamination. It is a promising organic-contaminant-cleaning technique with vast potential for development.

The low-pressure plasma cleaning technology is characterized by its efficiency, process controllability, *in situ* operation, and absence of secondary contamination. As a result, this technology has garnered significant attention from scholars, particularly for its application in cleaning organic contaminants from the surfaces of optical components. It has been shown to effectively restore the surface morphology of optical components with chemical coatings, thereby enhancing the components' optical transmittance and laser-damage resistance.^16,17^ Cuxart *et al.* employed a remote inductively coupled low-pressure RF plasma source (GV10x DownStream Asher) to clean carbon contaminants made of different carbon allotropes (amorphous and diamond-like carbon) from the surface of large-aperture optical components.^18^ After 6000 seconds of treatment, the thickness of the carbon contamination coating was reduced by 35%. Marinov *et al.* utilized hydrogen plasma to completely remove Polymethyl Methacrylate (PMMA) polymer contaminants from the surface of monolayer tungsten disulfide (WS_2_) coatings deposited by metal–organic chemical vapor deposition (MOCVD).^19^ Oxygen plasma was also used to remove contaminants from the surface of silicon carbide (SiC), significantly reducing its surface roughness from 1.090 nm to 0.055 nm.^20^ Marot *et al.* successfully applied 60 MHz RF plasma to clean the Edge Thomson scattering mirrors of the ITER project, achieving a cleaning uniformity of approximately 80%.^21^ Yadav *et al.* demonstrated the effectiveness of removing carbon contamination from gold mirror surfaces using ultraviolet light, RF plasma, and infrared laser exposure.^22^ Their comparative cleaning results showed that RF plasma could efficiently remove carbon contaminants from mirror surfaces and, without secondary contamination, restore the original optical performance of the optical components. Despite the extensive research on using low-pressure plasma for cleaning surface contaminants from optical components, the relationships between the plasma-discharge process parameters and cleaning effectiveness and the degree of influence of different process parameters on the cleaning outcomes remain unclear. Further research is needed to better understand and optimize these parameters for improved cleaning performance.

Although plasma cleaning technology has been widely applied in cleaning large-aperture optical components' surface chemical coatings, the mechanism of interaction between the plasma and contaminants during the cleaning process and the factors and mechanisms influencing the cleaning efficiency remain underexplored. These are areas that scholars in the field urgently need to investigate. Fraxedas *et al.* employed *in situ* X-ray photoelectron spectroscopy to characterize the changes in the surface structure of carbon contamination on ultra-high voltage (UHV) chamber surfaces during low-pressure inductively coupled plasma (ICP) cleaning. This study simulated the surface of optical components used in synchrotron accelerators, with free electron laser beamlines serving as a reference.^23^ Sun *et al.* utilized ReaxFF-based molecular dynamics simulations to investigate the mechanisms and parameters affecting plasma etching. Their work characterized both the fluorine atom etching process on single-crystal silicon and the mechanism by which fluorine atoms remove sub-surface damage in single-crystal silicon.^24^ Nie *et al.* combined molecular dynamics simulations and experimental validation to study the material removal and pit formation in the hydrogen and oxygen etching of dislocation regions, revealing the critical role of dislocation-induced crystal-structure destruction and symmetrically distributed stress in facilitating bond breaking and triggering etch pit formation.^25^ Liu *et al.* used ReaxFF molecular dynamics simulations to study the atomic removal mechanism in the plasma-assisted polishing (PAP) of single-crystal diamond (SCD) substrates.^26^ Many scholars have conducted in-depth research on the etching mechanisms of plasma-based surface processing, but there is still a knowledge gap on the mechanisms behind plasma cleaning technology. The primary processes of plasma cleaning of organic contaminants occur on nanosecond timescales and at atomic spatial scales, which makes direct, *in situ* observation at the surface inherently challenging. While advanced ultrafast spectroscopies can achieve nanosecond or faster temporal resolution, directly capturing the intermolecular chemical interactions between reactive plasma species and adsorbed organic contaminants at the surface on nanosecond time scales and under active plasma exposure remains highly demanding. This combination of stringent spatiotemporal requirements and a reactive, perturbed environment incentivizes the use of reactive molecular dynamics simulations, which can resolve the atomic-scale reaction pathways of plasma-surface interactions, thereby complementing experimental diagnostics.^27,30^ The studies mentioned above demonstrate that reactive molecular dynamics can simulate the atomic-scale micro-reaction processes of plasma, providing new insights into the study of plasma cleaning mechanisms. Combining simulation results with experimental data can offer rational and detailed explanations for the plasma reaction mechanisms and the factors influencing the cleaning efficiency, which represents an important research approach for further investigating the core mechanisms of plasma cleaning technology.

This study focuses on the chemical coating on the surface of optical components in intense laser systems, investigating the cleaning process and mechanisms of low-pressure plasma cleaning technology for organic contaminants. Firstly, a Langmuir probe was employed to study the effects of discharge power and gas pressure on the plasma potential, ion density, and electron temperature. Secondly, a combination of single-factor and orthogonal experiments was conducted to measure the cleanliness of the optical component surface and the recovery of its optical performance. Then, starting from the chemical reaction process between low-pressure plasma and organic contaminants, a Reactive Molecular Dynamics (RMD) model was constructed to simulate the cleaning process of organic contaminants under different bombardment energies and ion fluxes, thereby understanding their effects on the cleaning process. The microscopic mechanisms of the plasma and the organic contaminant interactions are compared, and the cleaning effect patterns derived from macro experiments are validated.

## 2. Experimental and simulation methods

### 2.1 Preparation of experimental samples

**Fig. 1:**
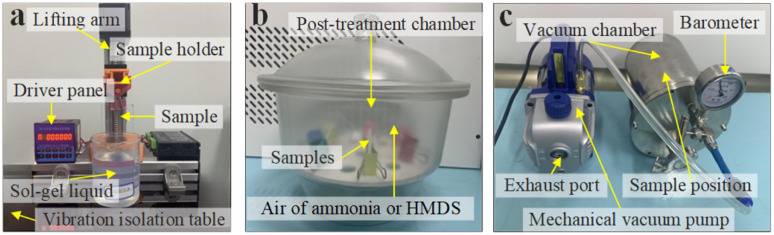
Experimental sample preparation equipment: (a) lifting coater to deposit chemical coatings on samples; (b) post-treatment of the coating with ammonia and HMDS; and (c) contamination process of the chemical coatings with DBP.

This study employed a dip-coating method to prepare chemical-coated fused silica samples using sol–gel SiO_2_ at 355 nm wavelength. The selection of 355 nm, driven by its advantages of short wavelength and high photon energy characteristics, addresses the extreme optical performance requirements of intense laser systems in cutting-edge applications.^31–33^ In this study, the chemical coating with a particle size of 29 nm SiO_2_ was prepared at 25 °C on a clean fused silica substrate with a pull-coating machine at an 85 mm min^−1^ pull speed. The equipment for the dip-pull coating machine is shown in Fig. 1(a), and the experimental parameters for the sample preparation are consistent with those of Li *et al.*^34^ The sample was lowered into the beaker until three-quarters of its height was submerged, after which it was left still for 2 minutes to ensure full contact between the colloid and the sample surface. Then, the sample was pulled out of the beaker at a constant speed and removed for post-treatment.^35^Fig. 1(b) shows the post-treatment of the chemical coating with ammonia and hexamethyldisilazane (HMDS), which involved placing the post-treatment reagents and samples in a sealed glass container for 24 hours.

Furthermore, organic contamination treatment is required in a vacuum environment to simulate the contamination of samples under the vacuum operating conditions of the intense laser system, as shown in Fig. 1(c). The chosen contaminant is dibutyl phthalate (DBP), selected because contamination monitoring of large-aperture, chemically coated optics in the laser facility consistently detects DBP in high relative abundance on working surfaces. DBP includes functional groups, such as aromatic, ester and aliphatic groups, that are also common among co-detected contaminants.^28^ In our previous work, the main components and relative contents of organic contaminants on the wall and in the air at different positions in the chirped pulse amplification system were detected by gas chromatography and mass spectrometry. The organic molecules were volatilized from potential sources, such as components and pump oil or dust produced by stray light irradiation of carbon-based materials.^29^ Thus, we use DBP as a practically representative index contaminant to study the oxygen-plasma cleaning of real optical surfaces.^30^ DBP is highly volatile under reduced pressure, and it easily adheres and causes contamination when it contacts surfaces, such as device walls or optical components. The sample was placed in a vacuum chamber, and a DBP solution was poured into an open container with a mechanical pump connected to the vacuum chamber to lower the chamber's pressure to 10 Pa. All experiments were conducted in a clean room to simulate the organic contamination environment found in ICF facilities.

### 2.2 Low-pressure plasma cleaning

**Fig. 2:**
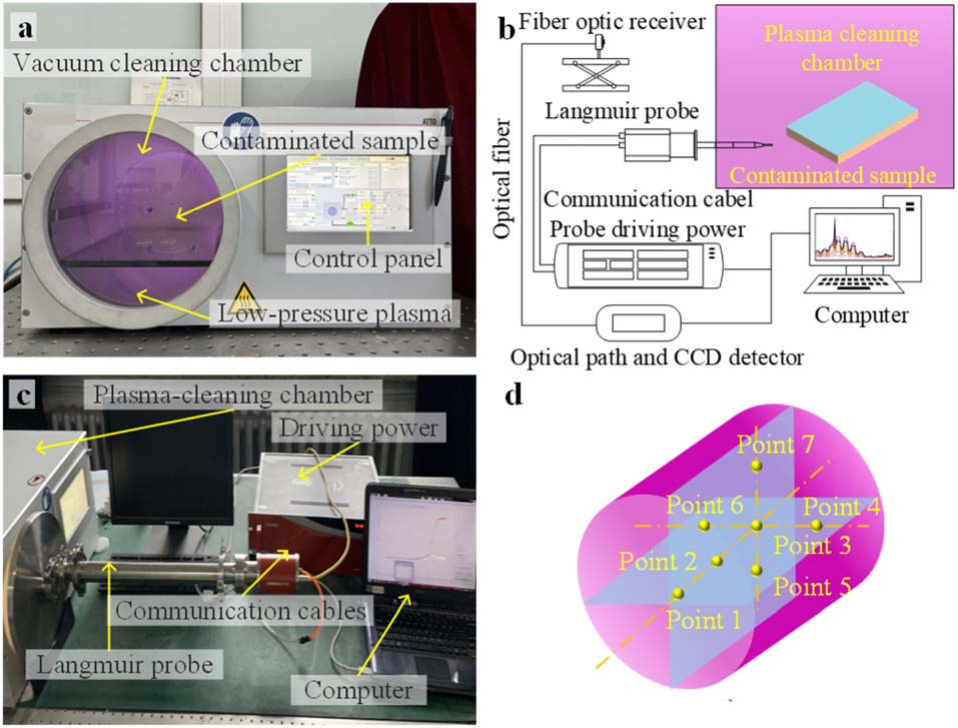
Low-pressure plasma cleaning equipment and diagnosis platform: (a) low-pressure plasma cleaning equipment; (b) schematic of the characteristics measurement platform of plasma discharge; (c) measuring equipment of the plasma discharge characteristics; and (d) schematic of the seven measurement positions using the Langmuir probe.

Fig. 2(a) illustrates the low-pressure plasma cleaning equipment used in the experiment, *i.e.*, a vacuum plasma cleaner of the Atto-LC-PC model. A diagnostic platform was established to explore the effects of pressure and power variations on the discharge characteristic parameters of plasma, as shown in Fig. 2(b) and (c). To obtain the plasma discharge characteristic parameters at different positions within the chamber, seven measurement points of the Langmuir probe were selected along the axial and cross-sectional directions, respectively, as depicted in Fig. 2(d).^36^ Also, in this study, a high-resolution spectrometer was employed to analyse the plasma emission spectra generated using high-purity oxygen and argon as excitation gases. The cleaning efficacy of the plasma treatment can be characterized by the surface wettability and transmittance of the samples.^27^ Due to the plasma-induced surface activation, wettability loses its ability to reliably indicate the surface cleanliness. Therefore, Raman spectroscopy was employed to detect the surface chemical composition, establishing a quantitative correlation between cleaning effectiveness and transmittance values through the analysis of the characteristic spectral intensities.

**Fig. 3:**
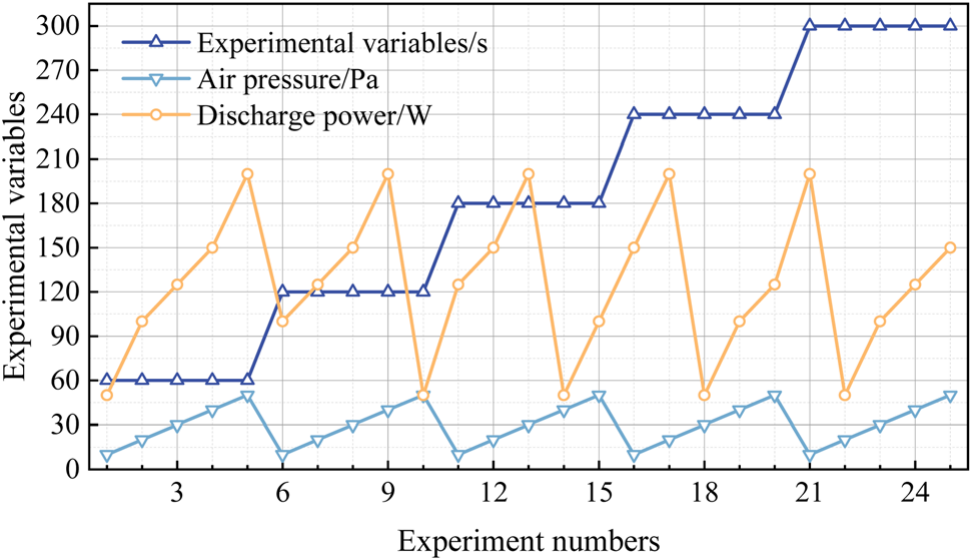
Illustration of the orthogonal experiment parameter matrix, where each factor (A: cleaning time, B: pressure, C: power) is varied over five levels. This design allows the evaluation of the independent effect of each factor *via* range analysis.

The process parameters that primarily influence the efficacy of low-pressure plasma cleaning are the cleaning time, discharge power, and working pressure. In this study, single-factor experiments were first conducted to analyse the effects of each process parameter, whose parameter design is shown in Table 1. Based on studies on low-pressure plasma discharge characteristics, the initial conditions of a 20 Pa working pressure and a 100 W discharge power were selected for the cleaning with duration variation. To avoid over-cleaning effects, a cleaning duration of 2 min was adopted as the baseline condition, as preliminary tests indicated that extending cleaning beyond 2 minutes under baseline conditions yielded no significant additional increase in the removal efficiency. Further, the choice of a 2-minute cleaning time can prevent the masking of the influence of experimental variables on the cleaning effectiveness (see Section 3.2 for further discussion). The ranges of the discharge power and working pressure were determined by discharge characteristic analyses. All experiments were conducted under 25 °C, with oxygen as the working gas. Transmittance measurements were performed by averaging the values from five random points on each sample. Then, orthogonal experiments were designed to evaluate the extent to which different process parameters affect the cleaning efficacy and to determine the optimal combination of process parameters for achieving the best cleaning results. Based on the results of the single-factor experiments, the selection of orthogonal experimental parameters is shown in Fig. 3. In the orthogonal experiments, the cleaning time (factor A), air pressure (factor B) and discharge power (factor C) each have 5 preset levels. This approach varies the factors simultaneously according to the orthogonal matrix, enabling us to quantify the influence of each factor using range analysis (presented later in Table 4). Additionally, in this work, “uncontaminated” denotes samples that were not intentionally exposed to DBP. Since large-aperture coated optics may adsorb trace airborne species (adventitious hydrocarbons, moisture) during routine transport and storage, the uncontaminated state should be regarded as the practical baseline rather than an ideal pristine surface. This behavior mirrors real facility conditions, where coated optics accrue trace airborne films during handling and storage. Plasma cleaning strategies are therefore judged by their ability to restore pre-contamination optical performance.

**Table 1:** Experimental parameter design in single-factor experiments (default experimental parameters: *P* = 100 W, *p* = 20 Pa, *t* = 2 min)

Cleaning time (*t*/min)	Discharge power (*P*/W)	Pressure (*p*/Pa)
1	50	10
2	100	20
3	125	30
4	150	40
5	200	50

### 2.3 Establishment of the finite element simulation model

**Fig. 4:**
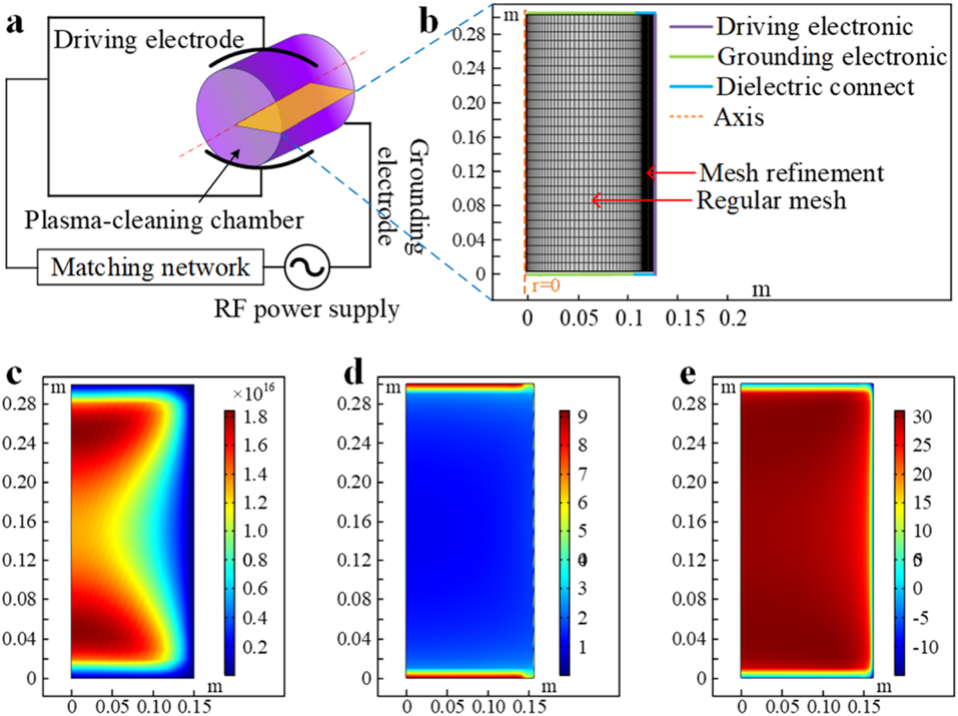
Finite element simulation model establishment: (a) schematic of the discharge of low-pressure plasma cleaning equipment; (b) boundary condition setting and meshing diagram of low-pressure plasma; (c) simulation result of the electron density when the air pressure is 10 Pa, the discharge power is 200 W, and the atmosphere is oxygen; (d) simulation result of the electron temperature; and (e) simulation result of the electric potential in space.

This study employs a fluid dynamics model to simulate the discharge process of low-pressure capacitively coupled RF plasma. The discharge characteristics of low-pressure plasma under different working pressures and discharge powers are calculated with the simulation model. The discharge schematic diagram of the low-pressure plasma cleaning equipment used in the experiment is shown in Fig. 4(a). The discharge chamber can be considered an axisymmetric structure. Consequently, symmetry assumptions during model construction significantly improve the computational efficiency. Given the sheath phenomenon near the electrodes, the mesh near the driving electrode should be refined to enhance the accuracy of the calculations. Fig. 4(b) illustrates the low-pressure plasma's boundary condition settings and mesh division.

The electron density and mean electron energy are computed by solving a set of drift-diffusion equations for the electron density and average electron energy, neglecting the convective effects of electrons induced by fluid motion.1
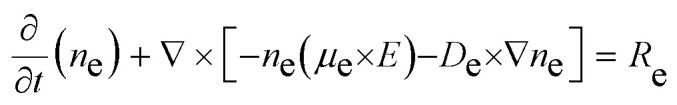
2
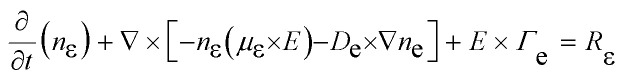
In the equations, ne represents the electron density, *µ*_e_ is the electron drift velocity, *E* is the electric field strength vector, *D*_e_ is the electron diffusion coefficient, *S*_e_ is the electron source term, *n*_ε_ is the electron energy, *µ*_ε_ is the electron energy drift rate, and *Γ*_e_ is the electron energy loss.

### 2.4 Establishment of the molecular dynamics model

To further explore the physical and chemical reaction mechanisms of energetic reactive particles in plasma interacting with organic contaminants, an RMD model for the interaction between energetic reactive particles and organic contaminants is established in this study. The model is constructed based on the ReaxFF force field, which aims to describe the stability and geometry of non-conjugated, conjugated, and radical-containing compounds.^37^ ReaxFF divides the system energy into contributions from various energy components, as shown in eqn (3).

3 *E*_system_ = *E*_bond_ + *E*_over_ + *E*_under_ + *E*_val_ + *E*_pen_ + *E*_tors_ + *E*_conj_ + *E*_vdWaals_ + *E*_coulomb_

where *E*_system_ represents the system energy, *E*_bond_ denotes the bond energy, *E*_over_ indicates the over-saturated bond energy, *E*_under_ refers to the under-saturated bond energy, *E*_val_ signifies the bond angle energy, *E*_pen_ represents the covalent bond correction energy, *E*_tors_ denotes the torsional angle energy, *E*_conj_ refers to the conjugation energy, *E*_vdWaals_ indicates the van der Waals interaction energy, and *E*_coulomb_ represents the electrostatic interaction energy.^38^

**Fig. 5:**
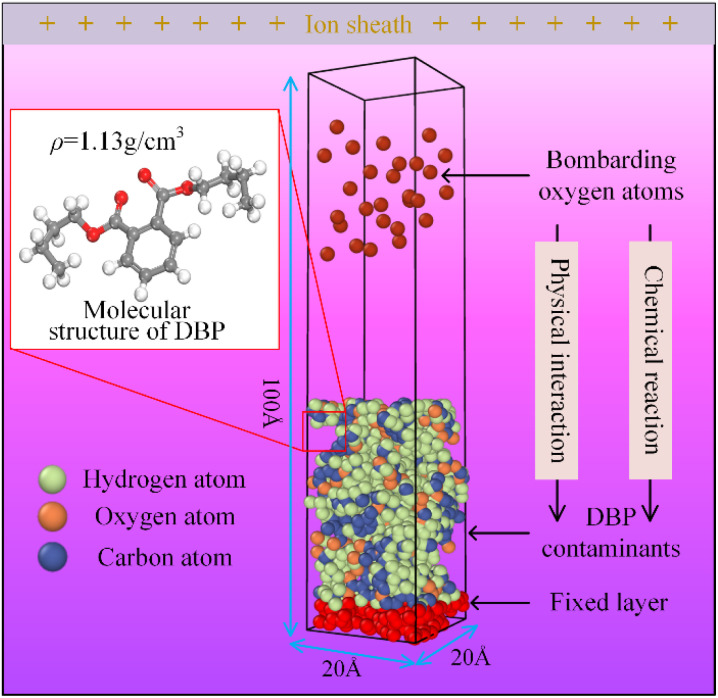
Molecular dynamics model of the reaction between plasma and organic contaminants.

Based on the spectral analysis results, the model selects energetic oxygen plasma as a representative of reactive particles. A vacuum simulation cell was constructed to replicate the vacuum working environment encountered during actual cleaning processes, as shown in Fig. 5. Since DBP is in a liquid state at room temperature, a liquid DBP contamination layer with a density of 1.13 g cm^−3^ was generated at the bottom of the simulation box. To prevent the overall movement of the contamination layer from affecting the calculation results, the bottom atoms of the contamination layer were fixed. Energetic oxygen plasma with initial velocities was randomly generated above the contamination layer. After establishing the model, the energy and flux of the energetic reactive particles and ambient temperature are adjusted by varying the initial velocity of the oxygen plasma, the insertion time interval of the oxygen plasma, and the temperature set by the Berendsen thermostat Table 2.

**Table 2:** Parameters of the reactive molecular dynamics model

Parameter	Number
Simulation cell dimensions	20 × 20 × 100 Å^3^
Number of atoms in the organic contaminant	1680
Number of oxygen plasma for irradiation	200
Ensemble	*NVT*
Boundary conditions	PPF
Timestep	0.5 fs
Total reaction duration	0.1 ns
Initial temperature	300 K
Temperature range	225–400 (K)
Velocity range of oxygen plasma	0.05–0.5 (Å^3^ fs^−1^)
Insertion time interval range of oxygen plasma	7.5–50 (fs)

## 3. Results and discussion

### 3.1 Characterization of low-pressure plasma discharge and investigation of reactive species

Studying low-pressure plasma discharge characteristics and reactive species is crucial for optimizing the cleaning process, enhancing the cleaning efficiency, and effectively removing organic contaminants. This study first provides a basis for setting and selecting process parameters in subsequent experiments. The research methodology involved simulation calculations at a working pressure of 10 Pa and a discharge power of 200 W, with oxygen as the working gas.

Eqn (1) was employed to calculate the electron diffusion coefficient, energy migration rate, and energy diffusion coefficient based on the electron mobility. The rate coefficients in the plasma chemical reaction system determine the electron source in the above equation. Eqn (2) was then employed to obtain the electron energy loss by summing the collision energy losses of all reactions and calculating the rate coefficients based on the cross-section data. The simulation results, shown in Fig. 4 (c)–(e), for a working pressure of 10 Pa, a discharge power of 200 W, and an oxygen atmosphere, result from this rigorous process. The analysis of the simulation results indicates that under these conditions, the electron density ranges from 1 × 10^15^ m^−3^ to 2 × 10^16^ m^−3^, with an inhomogeneous spatial distribution and two extreme values. The electron temperature ranges from 2 eV to 4 eV, with a relatively uniform spatial distribution. The spatial potential is within 35 V, with a relatively uniform spatial distribution.

**Fig. 6:**
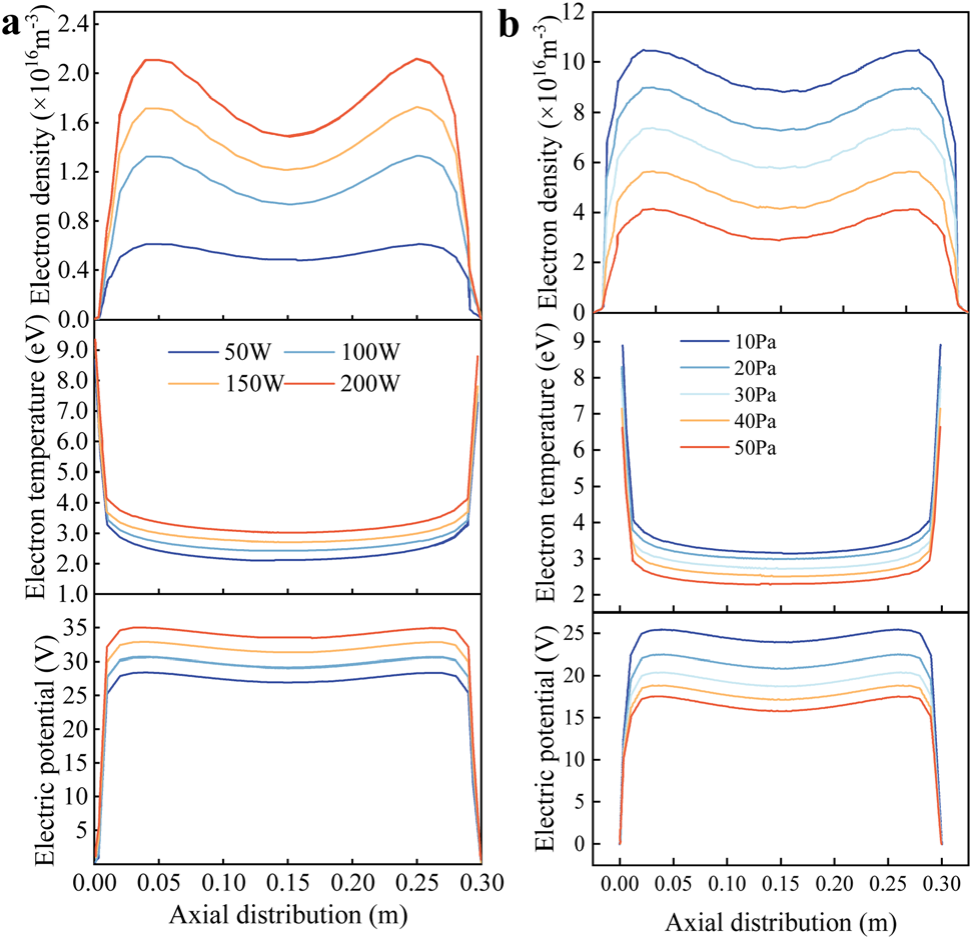
Finite element simulation results of the low-pressure plasma discharge process: (a) influence of the electron density, electron temperature and electric potential attributed to the discharge power; (b) influence of the electron density, electron temperature and electric potential attributed to pressure.

To analyse the influence of the discharge power on the characteristic parameters of plasma discharge, simulations were conducted at a working pressure of 20 Pa, with discharge powers set at 50 W, 100 W, 150 W, and 200 W. The average electron density, electron temperature, and spatial potential along the axis were obtained by integrating the simulation results over the time period and along the axis, as shown in Fig. 6(a). For a given discharge power, the axial electron-density profile is symmetric, peaking near the axis ends (close to the grounded electrode) and decreasing towards the center, and it shows the expected near-electrode sheath depletion at both ends (electron density falls rapidly to very low values within the narrow sheaths). This edge-depletion trend is present at all tested powers (50–200 W). This is due to the formation of a sheath in this region, causing electrons to move away and resulting in a maximum electron density in the central plasma region. Away from the sheaths, in the plasma bulk, the electron density increases monotonically with the discharge power because a higher RF power enhances ionization. In Fig. 6(a), the curves therefore have a similar shape across powers (sheath-edge drop at both ends and an axisymmetric “valley” shape), while their overall magnitude increases as power is raised. As the discharge power increases, electrons gain more energy, leading to more intense particle collisions, enhanced gas ionization, and, consequently, higher electron density. If the electrode configuration is altered, the electron density distribution along the axis exhibits an initial increase, followed by a decrease. In Fig. 6(a), the electron temperature peaks near the grounded electrode and reaches a minimum in the main plasma region, decreasing with reduced power. This is because, in a steady-state plasma, the electron temperature is only associated with the pressure and the system scale. The sheath electric field accelerates electrons away from this region at the two-axis ends (near the grounded electrodes). The closer to the electrode, the stronger the sheath electric field effect, the higher the electron energy, and thus the higher the temperature. Conversely, the spatial potential reaches an extreme value at the edge of the electrode after the formation of the sheath and increases with increasing discharge power. In radio-frequency capacitively coupled plasmas, the discharge power is typically controlled by varying the electrode voltage. When the electrode structure, gas composition, and pressure are constant, the impedance of the main plasma region is a fixed value. Therefore, the magnitude of the spatial potential is determined by the electrode voltage, which increases with increasing discharge power.

Under a discharge power of 100 W, the working pressure is set to 10, 20, 30, 40, and 50 Pa, sequentially. The average electron density, electron temperature, and spatial potential along the axis are obtained by integrating the simulation results over the time period and along the axis, as shown in Fig. 6(b). Changing the chamber pressure does not affect the spatial distribution of the low-pressure plasma characteristic parameters, and the distribution patterns are the same as those observed when analysing the influence of the discharge power on the plasma parameters. First, the electron density decreases with increasing pressure. This is because, with a limited power input into the main plasma region, an increase in pressure leads to more frequent electron-gas molecule collisions, thereby reducing the electron and ion densities. Second, the electron temperature decreases with increasing pressure. This is because the mean free path of ions is inversely proportional to the gas density. An increase in pressure results in a higher gas density, a shorter mean free path for ions, and a reduction in the ion and electron energies. Additionally, the gas pressure affects the particle energy loss at the surfaces of the two RF electrodes. An increase in pressure leads to greater particle energy loss, reducing the energy gained by the gas in the main plasma region and further lowering the electron temperature. Third, the spatial potential decreases with increasing pressure. This is because a decrease in the gas density reduces the capacitive impedance of the plasma region, leading to a smaller voltage drop across the electrodes and an increase in the spatial potential.

**Fig. 7:**
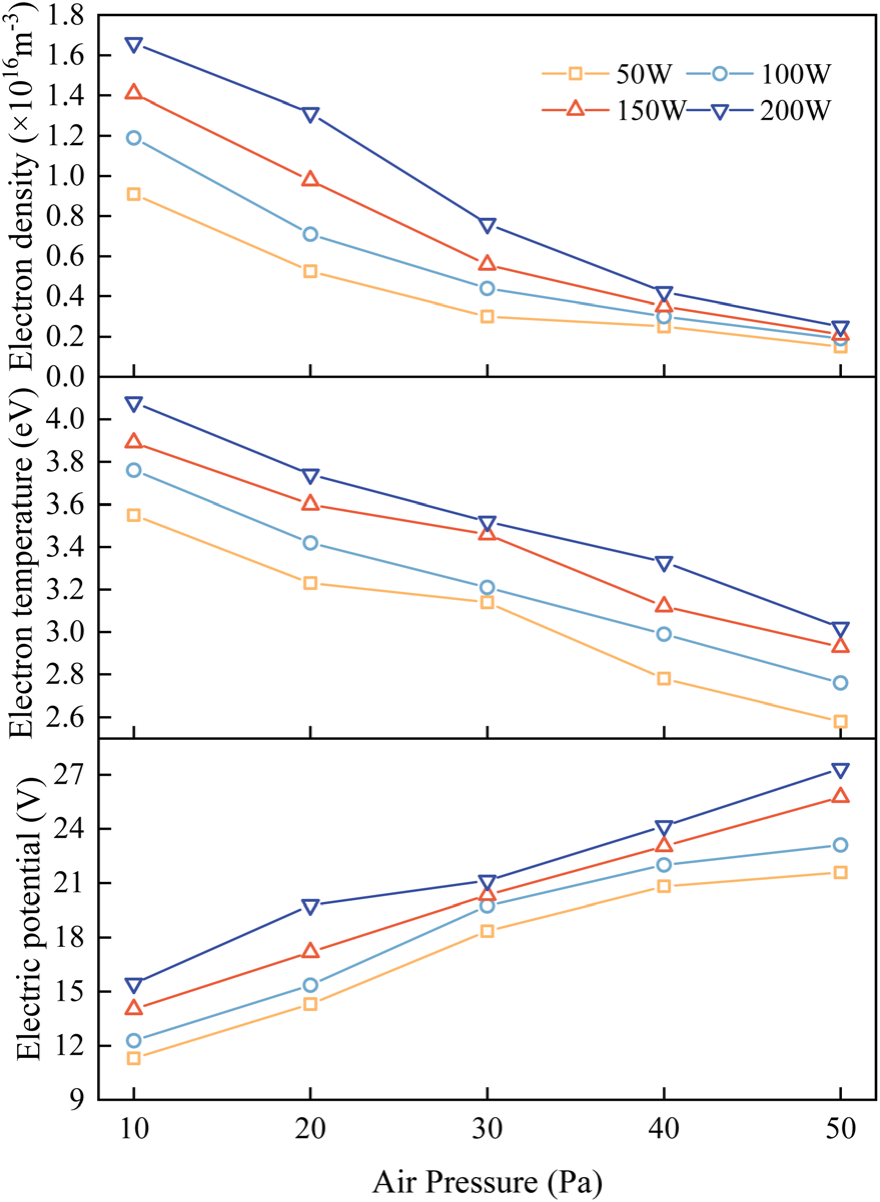
Single-point Langmuir probe measurement showing the measurement results of electron density, electron temperature and electric potential with different discharge powers and pressures.

When employing a single-point Langmuir probe for measurements, the effects of different process parameters on the plasma characteristic parameters can be obtained. Fig. 7 respectively depict the variations in the electron density, electron temperature, and spatial potential with pressure under different discharge powers. Experimental results demonstrate that within the set parameter range (working pressure of 10–50 Pa, discharge power of 50–200 W, and oxygen as the working gas), the following relationships hold: when the working pressure is constant, the electron density, electron temperature, and spatial potential all increase with increasing discharge power; conversely, when the discharge power is constant, the electron density, electron temperature, and spatial potential all decrease with increasing working pressure. The ranges of low-pressure plasma characteristic parameters obtained from the experiments are consistent with the simulation results, and the trends of the effects of the process parameters on the plasma characteristic parameters in experiments match those in the simulations. These results are also consistent with the experimental findings of Yin, who used a Langmuir probe to investigate the discharge characteristics of argon plasma.^15^ Therefore, it can be concluded that the finite element simulation model is reasonably set up and corresponds to the actual situation.

Measurements were taken at different points within the plasma chamber to investigate the spatial distribution of low-pressure plasma characteristic parameters and compare them with the theoretical simulation values. The analysis of discrepancies helps to ensure the accuracy of the simulation results. Table 3 compares the probe-measured data and the simulated values of the discharge characteristics under identical process parameters (discharge power of 100 W, working pressure of 20 Pa, and oxygen as the working gas). The results indicate that the theoretically calculated electron density is close but slightly lower than the measured values. In contrast, the calculated electron temperature and spatial potential are slightly higher than the measured values. This discrepancy may arise from the incomplete consideration of atomic collisions and secondary electron emission in the simulation process. Secondary electron emission caused by atomic collisions increases the electron density but leads to energy loss in electrons, thereby reducing the electron temperature. It also reduces the impedance of the plasma region, causing the spatial potential to rise. The spatial distribution of plasma characteristic parameters shows some differences between actual measurements and theoretical calculations regarding trends. This may be due to the gas flow within the chamber during actual discharge processes. Some errors are inevitable due to the need to maintain a constant internal pressure through continuous vacuum pumping, due to gas-tightness issues. However, the differences between the test regions are relatively small. A complete uncertainty analysis was not recorded for the probe dataset in Table 3. Accordingly, we restrict the comparison to an order-of-magnitude and trend-level assessment: the measured and simulated values are of the same order, and their axial/parametric trends coincide. Potential offsets likely reflect the probe's systematic error and model idealizations.

**Table 3:** Comparison of the measured data and theoretical calculations of the spatial distribution of discharge characteristics

Position	Measured values *n*_e_ (m^−3^)	Theoretical values *n*_e_ (m^−3^)	Measured values *T*_e_ (eV)	Theoretical values *T*_e_ (eV)	Measured values *V*_p_ (V)	Theoretical values *V*_p_ (V)
P-1	1.05 × 10^16^	1.034 × 10^16^	3.07	3.104	22.4	23.013
P-2	1.01 × 10^16^	1.0213 × 10^16^	3.01	3.114	21.9	22.561
P-3	1.05 × 10^16^	1.0112 × 10^16^	3.07	3.128	21.4	22.023
P-4	1.02 × 10^16^	1.0103 × 10^16^	2.98	3.128	20.9	21.862
P-5	1.03 × 10^16^	1.0103 × 10^16^	3.10	3.128	21.3	21.862
P-6	1.03 × 10^16^	1.0103 × 10^16^	3.03	3.128	22.3	21.862
P-7	1.06 × 10^16^	1.0103 × 10^16^	3.06	3.128	23.1	21.862

**Fig. 8:**
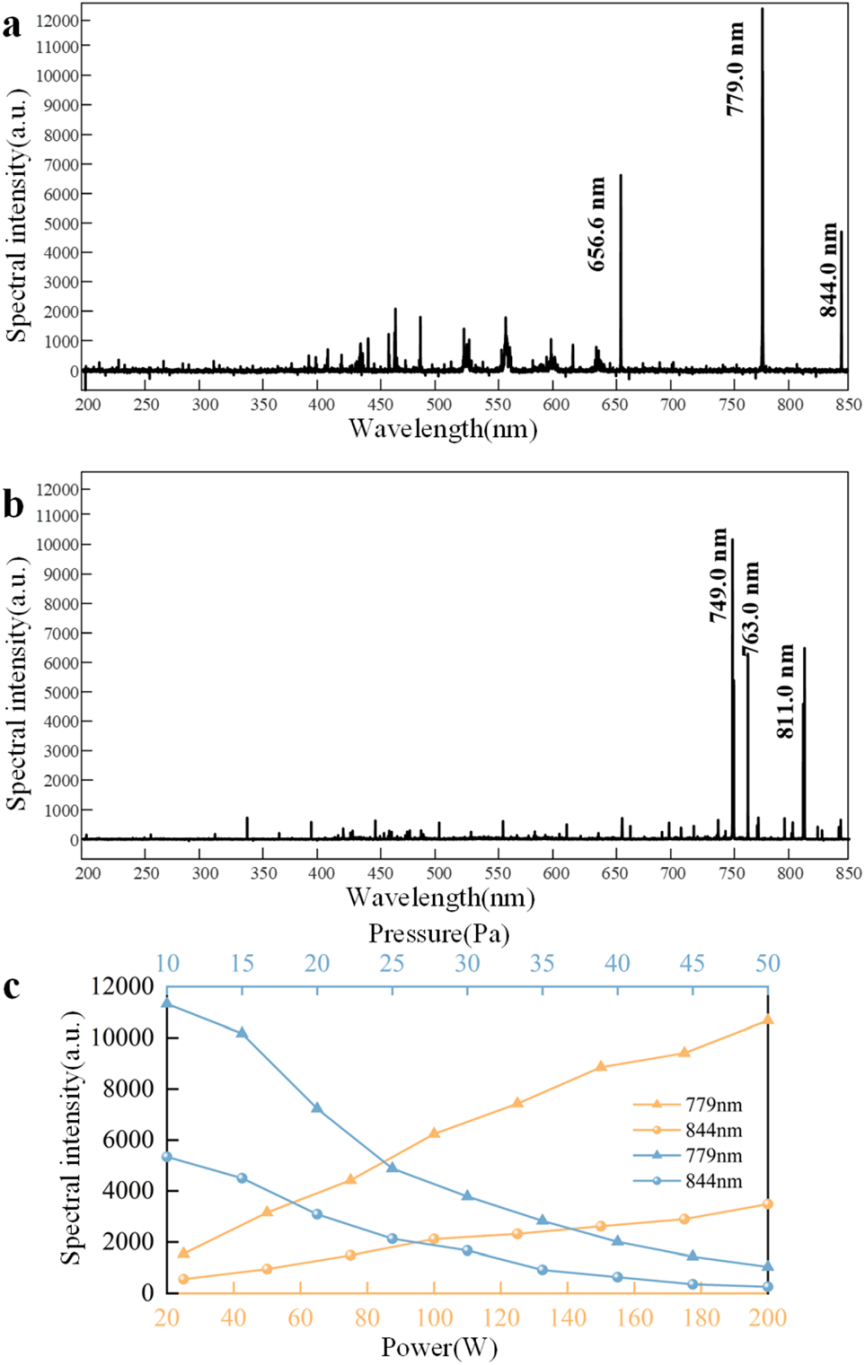
Emission spectrum of plasma: (a) oxygen plasma emission spectrum; (b) argon plasma generation spectrum; (c) curves of the intensity of different oxygen excitation characteristic spectra (with wavelengths of 779 nm and 844 nm, respectively) varying with power and pressure. The horizontal axis of the yellow curve, located at the bottom of the spectra, corresponds to the power variation. The horizontal axis of the blue curve, located at the top of the spectra, corresponds to the change in air pressure.

The analytical principle of the spectrometer is based on the absorption of the characteristic spectra of the analyte elements emitted by the light source by the ground-state atoms of the analyte elements in the sample vapor. The concentration of the analyte elements in the sample is determined by the degree of attenuation of the emission spectra.^39^ To identify the groups and ions excited during the low-pressure plasma cleaning process, experiments were conducted using oxygen and argon as the working gases, and the plasma was analysed using a spectrometer. Fig. 8(a) and (b) shows the emission spectra of the oxygen and argon plasma. When oxygen is used as the working gas, the main excited particles include oxygen atoms in the excited state with a wavelength of 779 nm, corresponding to the transition of energy levels 3s^5^s^0^ → 3p^5^p; oxygen atoms in the excited state with a wavelength of 844 nm, corresponding to the transition of energy levels 3s^3^s^0^ → 3p^3^p; and a small number of H_α_ atoms with a wavelength of 656.6 nm, corresponding to the transition of energy levels 3d^2^D → 2p^2^p^0^. The presence of these H_α_ atoms may be due to the water vapor in the plasma chamber or impurities in the gas. When argon is used as the gas source, the main excited particles include argon atoms in the excited state with a wavelength of 749 nm, corresponding to the transition of energy levels 4p^1^ → 4s^1^; argon atoms in the excited state with a wavelength of 811 nm, corresponding to the transition of energy levels 4p^9^ → 4s^5^; and argon atoms with a wavelength of 763 nm.

Fig. 8(c) illustrates the variation curves of the characteristic oxygen emission spectral intensities with power and pressure. It can be observed that there is a linear relationship between the power and gas excitation intensity. Within the range of 50 W to 200 W, the excitation intensity increases with the rise in the discharge power. In contrast, the pressure and gas excitation intensity exhibit an inverse functional relationship. The excitation intensity decreases with the chamber pressure within 10 Pa to 50 Pa. The spectral analysis results are consistent with the probe measurements and simulation calculations, which validate the probe's and spectral measurements' accuracy. The spectral intensities of various excited particles indicate that low-pressure plasma can excite argon and oxygen atoms, which interact with organic contaminants and the surface of the coating layer.

### 3.2 Influence of process parameters on the effectiveness of low-pressure plasma cleaning

**Fig. 9:**
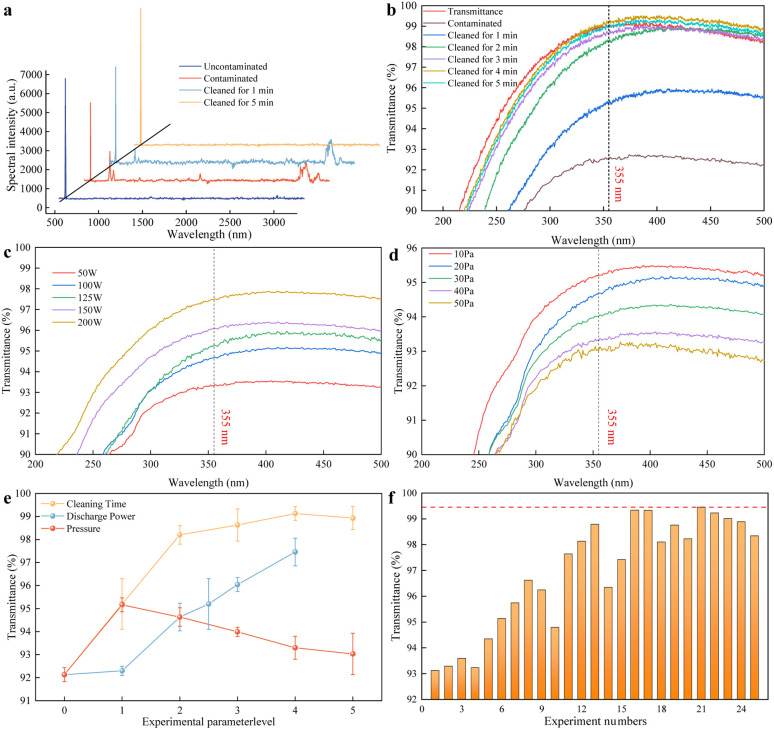
Experiment of plasma used to remove organic contaminants: (a) spectral intensity of samples under different conditions, which indicates that the transmittance equals the cleanliness of the surface of the samples; (b) transmittance of samples with different cleaning durations; (c) influence of discharge power on the cleaning effect; (d) influence of air pressure on the cleaning effect; (e) transmittance at 355 nm under variable parameters; (f) the best combination of process parameters for organic contaminant removal, where the red line indicates the best cleaning rate for all experiments.

A laser confocal Raman spectrometer was employed to detect the chemical composition of the optical components' surface. Samples were measured before contamination, after 24 hours of contamination with DBP, after 1 minute of low-pressure plasma cleaning, and after 5 minutes of cleaning. The Raman spectral analysis results are shown in Fig. 9(a). According to the literature, the characteristic peaks at 464 cm^−1^ and 799 cm^−1^ in the spectrum represent the asymmetric and symmetric stretching vibrations of the Si–O–Si bonds in the chemical coating, respectively. Comparing with the spectrum of the sample that had not been contaminated with organic substances, it can be seen that several characteristic peaks of functional groups appeared in the Raman spectrum after DBP contamination. The peaks at 670 cm^−1^ and 741 cm^−1^ represent the C–H bending vibration peak in the benzene ring and the C–C skeletal vibration peak in the benzene ring of the DBP molecular structure, respectively. The peak at 1729 cm^−1^ is caused by the stretching vibration of the carbon–oxygen double bond in the side chain of the DBP molecular structure. The broadening of the peaks in the range of 2800–3000 cm^−1^ is due to the stretching vibrations of C–H in the side chain of the DBP molecular structure, including symmetric, asymmetric, and non-planar stretching vibrations.

After one minute of low-pressure plasma cleaning, it can be observed that the intensities of several characteristic peaks corresponding to the benzene ring decrease. This indicates that the benzene rings in DBP have been dissociated by low-pressure plasma treatment, reducing their quantity and, thus, lowering the intensity of the characteristic peaks. The spectral signal of the C=O double bond stretching vibration peak is extremely weak, suggesting that the C=O double bonds in DBP are highly unstable and prone to dissociation. In contrast, the intensities of the C–H and CH_3_ peaks do not weaken like the other typical DBP peaks; instead, they increase compared to their intensities before cleaning. This is attributed to the dissociation of DBP's benzene rings and side chains into smaller hydrocarbon molecules. The transmittance curve of the sample in Fig. 9(b) indicates that the optical element has a transmittance of 92.13% after 24 hours of contamination. After one minute of cleaning, the transmittance recovers to 95.20%, which is lower than the value before contamination. Moreover, the characteristic peaks of DBP in the Raman spectrum have weakened but not completely disappeared, indicating that the contaminants on the sample surface have not been completely removed.

After five minutes of low-pressure plasma cleaning, the sample transmittance was restored to 99.22%, consistent with the initial state. At this point, the characteristic peaks of DBP in the Raman spectrum had all disappeared, indicating that the organic contaminants on the sample surface had been completely removed. It should be noted that under normal circumstances, SiO_2_-fused quartz coated with an antireflective layer can achieve a transmittance of up to 99.9% for light at a wavelength of 355 nm. However, the alcohol content of the adhesive used in the experiment may have evaporated during transportation and repeated use, leading to decreased adhesive quality and reduced transmittance. This does not affect the experimental results as long as the transmittance is restored to the initial level. Since Raman spectroscopy cannot quantify the amount of surface contaminants using spectral intensity, changes in transmittance can be used to indirectly quantify the amount of organic contaminants on the sample surface. Therefore, in subsequent experiments, the cleaning effectiveness can be quantitatively evaluated through the transmittance of the samples.

The single-factor method was employed to analyse the impact of cleaning time on the cleaning effectiveness. According to the experimental methods in Section 2.2, the influence of the cleaning time, discharge power, and working pressure on the transmittance was obtained. The transmittance variation curves of the samples cleaned under different working conditions are shown in Fig. 9(c) and (d), and the transmittance variation curves at the 355 nm wavelength are shown in Fig. 9(e). First, it can be seen from the curves that the transmittance of the samples increases rapidly at first, then more slowly, and eventually plateaus as the low-pressure plasma cleaning time increases, until it stabilizes. This indicates that low-pressure plasma is highly effective in cleaning organic contaminants. As time increases, the change in the surface cleanliness gradually stabilizes, as most of the contaminants on the surface have been effectively removed. From the study on the effect of cleaning time on cleaning effectiveness, it is known that after 3 minutes of cleaning, the change in transmittance is no longer significant, indicating that 3 minutes is sufficient to clean the experimental samples. To avoid the influence of other experimental variables on the cleaning effectiveness due to excessive cleaning duration, a cleaning time of 2 minutes was used in subsequent experiments. Next, the influence of the discharge power on the cleaning effectiveness was analyzed. According to the study on low-pressure plasma discharge characteristics in Section 3.1, the higher the discharge power, the higher the concentration of reactive particles and the spatial potential. Thus, the discharge power is crucial for achieving high cleaning effectiveness. First, it can be seen from the transmittance curve at the 355 nm wavelength that even with the maximum discharge power of 200 W, the transmittance of the cleaned samples has not yet returned to the initial value, indicating that there are still residual contaminants on the surface of the samples and no over-cleaning state has occurred. This means that over-cleaning will not mask the influence of the discharge power on the cleaning effectiveness. Under the same conditions, the transmittance of the samples increases linearly with increasing discharge power. According to the study on discharge characteristics in Section 3.1, an increase in power will lead to an increase in electron density and spatial potential. It can be reasonably speculated that the increase in the electron density and spatial potential can enhance the cleaning effectiveness of organic contaminants, as the removal mechanism of organic contaminants involves high-energy electrons and reactive particles interacting with organic contaminants through physical/chemical reactions. This result is consistent with the experimental results obtained by Wang using hydrogen plasma for *in situ* cleaning.^40^ Similarly, to investigate the influence of the working pressure on the cleaning effectiveness, experiments were conducted at a discharge power of 100 W and a cleaning time of 2 min, with oxygen as the working gas to prevent over-cleaning from masking the influence of experimental variables on the cleaning effectiveness. The transmittance variation curve at the 355 nm wavelength shows that under the same cleaning parameters, the transmittance of the samples decreases with increasing pressure, indicating that the amount of contaminants removed from the surface of the samples decreases, and the cleaning efficiency reduces as the pressure increases. According to the study on discharge characteristics in Section 3.1, an increase in pressure leads to a higher neutral gas density in the discharge region. This, in turn, causes more frequent electron-neutral collisions and gas impedance, which actually reduce the electron density and the plasma potential. It can be inferred that the number and energy of the reactive particles mainly involved in removing organic contaminants are reduced, leading to a decline in the cleaning effectiveness.

During the experimental process, it was observed that after prolonged or high-power cleaning, the transmittance of the optical components exhibited a downward trend following the peak transmittance achieved at the clean state. Based on this observation, a hypothesis is proposed: after the low-pressure plasma has completely removed the organic contaminants adhered to the chemical coating on the optical components, reactive species and high-energy electrons continue to bombard the chemical coating, inducing physical/chemical reactions with the coating. This process may lead to structural damage of the chemical coating on the optical components, thereby reducing the laser-induced damage threshold and degrading the optical performance. Considering the porous nature of the chemical coating, reactive particles may become embedded in the voids of the coating after bombardment or may displace the original particles within the coating, compromising the chemical coating's stability and resulting in a decline in optical performance. Therefore, future research could focus on investigating the impact of low-pressure plasma cleaning technology on the porous structure of chemical coatings and on how to optimize the process parameters of plasma cleaning technology to prevent secondary damage to the chemical coatings on optical components. Notably, beyond 355 nm, we observe that the 5 min curve lies slightly above the uncontaminated curve. This arises because the uncontaminated sample, while free of intentional DBP, can carry weakly bound adventitious films during handling, transport, and storage. Extended plasma exposure removes/oxidizes these residues, yielding a minor transmittance gain at specific wavelengths. Additionally, the microscale spatial non-uniformity of the coating and the measurement scatter can produce small ordering differences among the 1–5 min curves (*e.g.*, 4 min > 5 min) that are within the uncertainty level. These subtle effects do not affect the main conclusion that 4–5 min cleaning restores the optics to a saturated, near-baseline transmittance. It is worth noting that in this study, we employed continuous plasma exposure for cleaning. Investigating a pulsed plasma approach (short plasma bursts with intermittent rests) could be a valuable direction for future research, as it may affect the cleaning efficiency and help manage the thermal or physical stress on sensitive optical coatings.

To effectively control the cleaning quality and achieve the desired cleaning effect, it is necessary to investigate the relative importance of the adjustment of each process parameter and the optimal combination of cleaning process parameters during the cleaning process. According to the orthogonal experimental design in Section 2.2, the orthogonal experimental results are shown in Fig. 9(f).

The orthogonal experimental results were analysed using the range analysis method, and the results are shown in Table 4. In the table, Ni represents the sum of the experimental data at a certain level of a factor, and Mi represents the corresponding average value. The transmittance of the samples at the wavelength of 355 nm was obtained through orthogonal experiments. The difference between the maximum and minimum transmittance values under each process parameter was calculated to evaluate the dispersion degree of the data. A higher dispersion degree indicates a greater influence of the process parameter on the transmittance. As can be seen from the table, the order of the range sizes is RA > RC > RB, which means that the factors influencing the results in descending order are cleaning time, discharge power, and working pressure. Moreover, the best cleaning effect in the orthogonal experiment is achieved with the optimal process parameter combination, A5 B1 C5. The optimal cleaning effect is achieved with a cleaning time of 300 s, a discharge power of 200 W, and a pressure of 10 Pa.

**Table 4:** Orthogonal experimental data analysis

Result	Experimental number	Cleaning time A	Pressure B	Discharge power C
Transmittance %	N1	467.59	484.69	481.59
	N2	478.54	485.73	483.42
	N3	488.34	486.12	484.1
	N4	493.56	483.27	485.67
	N5	494.92	483.14	488.18
	M1	93.52	97.22	96.32
	M2	95.71	97.15	96.68
	M3	97.67	97.94	96.82
	M4	98.71	96.65	97.13
	M5	98.98	96.63	97.64
	Range *R*	5.47	0.60	1.32
	Order of significance	RA > RC > RB		
	Optimal combination	A5 B1 C5		

### 3.3 Physicochemical mechanism of the low-pressure plasma cleaning of organic contaminants

**Fig. 10:**
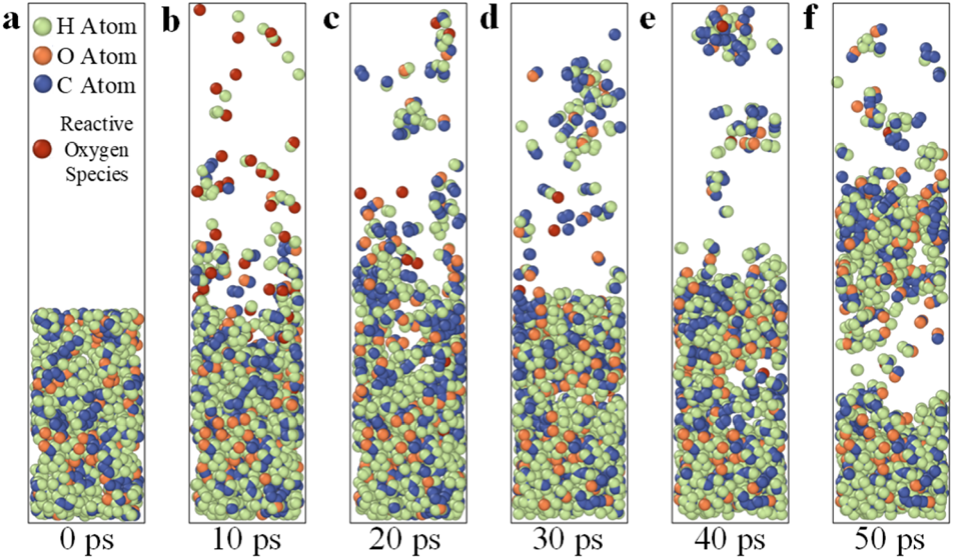
Snapshot of the layer-by-layer removal mechanism of DBP molecules under the physical and chemical actions of energetic reactive oxygen plasma and the simulated calculation results of the products; the green balls indicate the H atoms, the orange balls indicate the O atoms, the blue balls indicate the C atoms and the red balls indicate the reactive oxygen species. (a) DBP molecules removal after 0 ps plasma treatment; (b) DBP molecules removal after 10 ps plasma treatment; (c) DBP molecules removal after 20 ps plasma treatment; (d) DBP molecules removal after 30 ps plasma treatment; (e) DBP molecules removal after 40 ps plasma treatment; (f) DBP molecules removal after 50 ps plasma treatment.

Based on the reactive molecular dynamics model mentioned in Section 2.4 and according to previous research results, the environmental temperature was set at 300 K, the velocity of energetic reactive oxygen plasma at 0.3 Å ps^−1^, and the insertion time interval at 25 fs, affording an oxygen plasma energy of 74.7 eV and an oxygen plasma flux of 1 × 10^31^ m^−2^ s^−1^.^30^ Simulations were then conducted under these conditions. Fig. 10(a)–(f) illustrate the gradual dissociation and removal of organic contaminants under the continuous irradiation of energetic reactive particles. The reaction process is described from two aspects: physical and chemical interactions. As shown in Fig. 10(b), the surface DBP molecules first interact physically and chemically with oxygen plasma. Under the bombardment of energetic reactive oxygen plasma, hydrogen atoms with a relatively small mass are directly ejected from the contamination layer as hydrogen ions due to physical impacts. Subsequently, surface DBP molecules undergo chemical reactions with reactive oxygen plasma. Given the relative stability of the benzene ring, which makes it less reactive, the carbon, hydrogen, and oxygen atoms in the side chains outside the benzene ring first react with the reactive oxygen plasma, resulting in the dissociation of small molecules, such as H_2_O, OH^−^, H_2_, CHO^−^, and CO. Fig. 10(c) and (d) show that as the irradiation of reactive oxygen atoms progresses, deeper DBP molecules begin to participate in the reactions. Even DBP molecules that have not undergone chemical reactions start to detach from the contamination layer due to the physical bombardment of oxygen plasma. However, since DBP is in a liquid state at room temperature, the unreacted DBP molecules re-deposit and are not removed. Unlike the case in the initial reaction stage, the energetic reactive oxygen plasma, driven by its velocity, can penetrate deeper into the contamination layer. Therefore, reactive oxygen plasma can simultaneously undergo chemical reactions with the surface and deeper DBP molecules. As the chemical reactions progress, even the stable benzene ring dissociates, forming long-chain hydrocarbons, indicating further removal of organic contaminants. Meanwhile, due to the extremely high activation energy of the energetic reactive oxygen atoms in the plasma, DBP molecules and their dissociation products are further oxidized, generating substances such as H_2_O_2_, CO_2_, C_2_O_2_^2−^, CH_2_O, and C_2_H_2_O_2_^−^. Fig. 10(e) and (f) show that the DBP molecules continue to collide with oxygen plasma, and most of the deeper DBP molecules dissociate into small hydrocarbon molecules. On the one hand, the contamination-layer molecules continually gain upward velocity due to the recoil force. On the other hand, the small molecular products dissociated from the deeper contaminants continuously diffuse upward in the vacuum environment. Under the combined effect of these factors, contaminant molecules are observed to detach from the contamination layer in clusters, achieving further removal of organic contaminants. This is consistent with the experimental apparatus conditions described in Section 3.2. In the experimental setup, the vacuum pump continuously operates to maintain a vacuum environment, extracting the small molecules generated from the dissociation of contaminants in the cleaning chamber. The physical bombardment and chemical reaction of energetic reactive oxygen plasma loosen the DBP contamination layer, preventing it from adhering to itself, thereby enabling the layer-by-layer removal of organic contaminants.

**Fig. 11:**
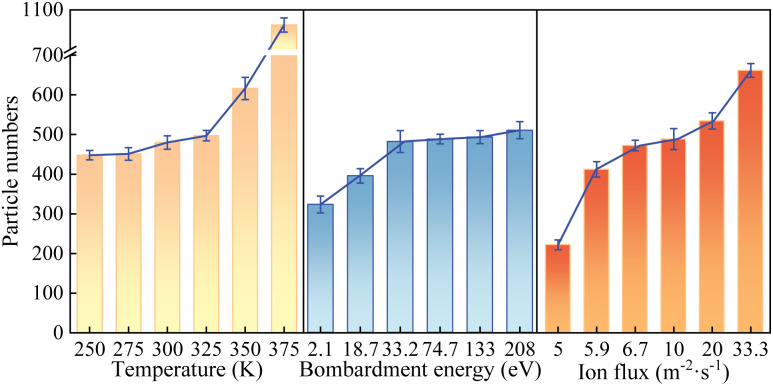
Statistics of the variation in the number of contaminant particles removed in the plasma cleaning simulation experiment with each experimental parameter.

Experiments were conducted by varying the energy, flux, and environmental temperature of the energetic reactive oxygen plasma to further investigate the effects of different plasma characteristics on the removal efficiency of organic contaminants. The results of these experiments were then correlated with the plasma discharge characteristics and process parameters to elucidate the underlying mechanisms influencing the cleaning outcomes. The gradient experimental parameters are summarized in Table 5. The simulation results of the electron density and temperature were described in Section 3.1. In order to accelerate the reaction between the plasma and the organic contaminants in a short period, higher plasma density and energy values were chosen for the simulation experiment, without affecting the study of the plasma degradation mechanism of organic contaminants. Under identical conditions, simulations were performed, and the number of remaining atoms in the contamination layer was counted after the simulations. The number of removed contaminant atoms was calculated based on this data, which served as a criterion for evaluating the cleaning effectiveness. The experimental results are shown in Fig. 11. According to the simulation results in Fig. 11, an increase in the environmental temperature, plasma energy, and plasma flux led to a rise in the number of removed organic contaminant atoms.

**Table 5:** Various parameters designed for the molecular dynamics simulation experiment to investigate their influence on the cleaning effectiveness (default experimental parameters: *E* = 33.2 eV, *F* = 10^31^ m^−2^ s^−1^, *T* = 300 K)

Temperature *T* (K)	Bombardment energy *E* (eV)	Ion flux *F* (×10^30^ m^−2^ s^−1^)
250	2.1	5.0
275	18.7	5.9
300	33.2	6.7
325	74.7	10.0
350	133.0	20.0
375	208.0	33.3

The mechanisms through which different plasma characteristic parameters influence the cleaning effectiveness are analysed from the perspective of the reaction mechanisms. First, temperature is a macroscopic manifestation of molecular thermal motion. An increase in the ambient temperature increases the average kinetic energy of the organic contaminant molecules and the energetic reactive oxygen plasma within the simulation box. The proportion of high-energy molecules increases, the velocity of molecular motion increases, and the frequency of molecular collisions per unit time rises, directly resulting in an increased reaction rate. Concurrently, the elevation in temperature may alter the reaction pathways for the removal of organic contaminants by reactive oxygen plasma. This would enable the reactions to proceed through more efficient routes, thereby increasing the reaction rate, leading to the removal of more organic contaminants within the same time frame. Second, increased plasma energy enhances the physical bombardment effect of reactive oxygen plasma on organic contaminants. Surface organic contaminants are more likely to be sputtered out, increasing the probability of contact with oxygen plasma and facilitating subsequent reactions. Moreover, reactive oxygen plasma can penetrate the contamination layer through bombardment, allowing it to react with surface and deeper contaminant molecules, further increasing the reaction rate. On the other hand, higher plasma energy implies a greater proportion of highly reactive particles, with more reactive oxygen plasma participating in the cleaning reactions, thereby accelerating the reaction rate. Lastly, increased plasma flux enhances the physical bombardment effect of reactive oxygen plasma on organic contaminants. An increase in plasma flux is equivalent to an increase in the number of reactive oxygen species participating in the cleaning reactions per unit time, which promotes the cleaning reaction. From the perspective of plasma discharge characteristics, an increase in the bombardment energy and ambient temperature is equivalent to an increase in the electron temperature, while an increase in the ion flux is equivalent to an increase in the electron density. According to the results analysed in Sections 3.1 and 3.2, both an increase in the electron temperature and an increase in the electron density can enhance the effectiveness of plasma cleaning of organic contaminants. Therefore, the experimental results and the results of molecular dynamics simulations can validate each other, demonstrating that establishing the molecular dynamics model has certain merits. Additionally, the simulation sequence mirrors the FTIR/XPS trajectories we reported for air/oxygen plasmas on coated optics (depletion of aliphatic/aromatic bands and temporary enrichment of oxygenated carbon before full removal).^30,34^ In parallel, the predicted kinetic trends align with our OES-based capability metric, in which O-atom emission increases with power and decreases with pressure, in line with the cleaning efficacy. Finally, the low-pressure, minute-scale process window that yields fast removal without damage in our experiments is reproduced mechanistically by the simulations, which rationalizes why over-exposure can eventually diminish optical performance.^41,42^ Since the simulated pathways and rate controls agree with our prior FTIR/XPS/OES signatures and with the demonstrated transmittance/LIDT recovery, the model provides a sound basis for parameter selection and can help reduce experimental iteration in future *in situ* cleaning campaigns.

## 4. Conclusions

In summary, this study, through experimental and simulation approaches, elucidated the factors and mechanisms influencing the effectiveness and efficiency of plasma cleaning for the removal of organic contaminants from optical component surfaces while clarifying the microscopic mechanisms underlying the cleaning process. Initially, a capacitive-coupled macroscopic model of the low-pressure plasma cleaner was constructed using the finite element method. This yielded the spatial distribution of discharge characteristic parameters, such as electron density, electron temperature, and spatial potential. Langmuir probe experiments were then employed to investigate the influence of process parameters on plasma characteristics, clarifying the types of reactive particles involved in the actual cleaning process. As the discharge power increased, the electron density, electron temperature, and spatial potential all rose, while with increasing pressure, these parameters decreased. Emission spectra indicated that the primary reactive particles excited by oxygen plasma were oxygen atoms at wavelengths of 779 nm and 844 nm, while those excited by argon plasma were argon atoms at wavelengths of 749 nm, 763 nm, and 811 nm. Subsequently, single-factor experiments demonstrated the high efficiency of plasma cleaning technology in removing organic contaminants adhered to the chemical coatings on optical components. Orthogonal experiments revealed the impact of process parameters on the plasma cleaning effectiveness and enabled parameter optimization. Increasing the cleaning time and discharge power enhanced the plasma cleaning efficiency, with higher efficiency observed at a discharge power of 200 W and a pressure of 10 Pa. The order of influence of the process parameters on the cleaning effectiveness was cleaning time, discharge power, and working pressure. Finally, molecular dynamics simulations were adopted to establish a microscopic model of the interaction between the low-pressure plasma and organic contaminants. Through the simulations, the microscopic reaction mechanisms of the reactive particles with the organic contaminants on the chemical coating surface during the plasma cleaning were investigated. High plasma energy, plasma flux, and temperature values facilitated the decomposition and removal of contaminants, providing theoretical guidance for subsequent process experiments. The study also highlighted potential areas for further exploration regarding the porous structure of the chemical coating on large-aperture optical components in high-energy laser systems.

## Author contributions

Zixu Wang: data curation (equal); formal analysis (equal); investigation (equal); methodology (equal); software (equal); visualization (equal); writing – original draft (equal); writing – review & editing (equal). Yuhai Li: funding acquisition (equal); methodology (equal); project administration (equal); supervision (equal); writing – review & editing (equal). Peng Zhang: conceptualization (equal); funding acquisition (equal); project administration (equal); resources (equal); supervision (equal). Fei Wang: investigation (equal); methodology (equal); validation (equal). Laixi Sun: formal analysis (equal); resources (equal). Qingshun Bai: resources (equal); supervision (equal). Mingzhi Zhu: conceptualization (equal); project administration (equal); supervision (equal). Baoxu Wang: funding acquisition (equal); supervision (equal).

## Conflicts of interest

There are no conflicts to declare.

## Supplementary Material

RA-015-D5RA05699C-s001

## Data Availability

The data supporting this article have been included as part of the supplementary information (SI). Supplementary information is available. See DOI: https://doi.org/10.1039/d5ra05699c.
