# Supplementary material for: Low-pressure plasma cleaning of organic contamination from chemically coated surfaces: a study combining experimental and molecular dynamics studies
Source: RSC Adv. 2025 Nov 18;15(53):45122–35. doi: 10.1039/d5ra05699c (PMC12624504; doi:10.1039/d5ra05699c)
Supplement: RA-015-D5RA05699C-s001 [file RA-015-D5RA05699C-s001.pdf]

Supplementary Information (SI) for RSC Advances.
This journal is © The Royal Society of Chemistry 2025

Low-pressure Plasma Cleaning of the Chemical Coating Surface with Organic Contamination: A Study Combining Experimental and Molecular Dynamics

Zixu Wang, [a] Yuhai Li, *[b] Peng Zhang, *[c,d] Fei Wang, [b,c] Laixi Sun, [b] Qingshun Bai, [c] Mingzhi Zhu *[a] and Baoxu Wang [a]

The supplementary information includes the lammps runtime environment Settings, runtime condition Settings and runtime code designed in the paper.

```
dimension          3
units              real                        # Energy: eV Distance: Angstrom
Mass: Kg Times: picosecond
boundary           p p f                        # p = periodic, f= fixed
atom_style         charge                      # Classical particles

read_data     120DBP.data

variable num_tep equal 250.0
variable num_vol equal 0.10
variable num_mol equal 100

region bot block INF INF INF INF -30 -25 units box
group    bot_f region bot

group oil type 1 2 3 4 5 6 7
pair_style          reaxff    NULL #lmp_control
#pair_style          reaxff    lmp_control
pair_coeff          * * ffield.reax.cho    C C O O C C H O
#fix                   QEQ all qeq/reaxff 1 0.0 20.0 1.0e-1 reaxff

change_box              all z delta 0 50 units box
#change_box              all z delta -10 50 units box
#change_box              all x delta -10 10 units box
#change_box              all y delta -10 10 units box

region                  top block -5 5 -5 5 55 65 units box
group                   top type 8
```

```
#pair_style            reaxff    NULL #!!!!!!lmp_control
#pair_coeff            * * ffield.reax.CHO    C C O O C C H O
#fix                    QEQ all qeq/reaxff 1 0.0 20.0 1.0e-2 reaxff


neighbor              2 bin
#neigh_modify        delay 0 every 10 check yes
neigh_modify        every 10 delay 0 check no
timestep        0.25

fix    Set bot_f setforce 0.0 0.0 0.0

#!!!!!!setting
#comm_style    tiled
#balance         0.9 rcb
thermo           100

#dump              1 all atom 100 mini.xyz
#minimize        1e-8 1e-8 10000 10000
#undump            1


thermo_modify lost ignore flush yes
fix                    1 all nvt temp ${num_tep} ${num_tep} 10.0
fix                    5 all qeq/reaxff 1 0.0 10.0 1e-6 reaxff #可以跑
#fix                    5 all qeq/reaxff 1 0.0 20.0 1e-6 reaxff
fix                    2 all temp/berendsen ${num_tep} ${num_tep} 100.0
fix                    3 top nve


fix                        4  top  deposit  1000  8  ${num_mol}    12345  region  top  vz  -
${num_vol} ${num_vol}
fix                    zwalls all wall/reflect zlo EDGE


dump             dumpfil all custom 200 ${num_tep}-V${num_vol}-M${num_mol}.data
id type x y z
#dump             3 all custom 500 dump.atom.* id type q x y z

run             500000
```
